# Wearable Sensor-Based Analysis of Punch Acceleration and Plantar Pressure Distribution in Boxing

**DOI:** 10.3390/s26092707

**Published:** 2026-04-27

**Authors:** Liwa Sha, Wen Hsin Chiu

**Affiliations:** 1 Department of Sports Training, Jilin Sport University, No. 2476, Ziyou Road, Changchun 130022, China; liwa-sha@m111.nthu.edu.tw; 2 Department of Education and Learning Technology, National Tsing Hua University, No. 521, Nanda Rd., Hsinchu City 300, Taiwan; 3 Department of Kinesiology, National Tsing Hua University, No. 521, Nanda Rd., Hsinchu City 300, Taiwan

**Keywords:** wearable sensors, boxing biomechanics, plantar pressure, punch acceleration, kinetic chain

## Abstract

**Highlights:**

What are the main findings?

- Professional boxers showed higher punch acceleration and forefoot loading than amateurs.
- Forefoot plantar pressure was positively associated with punching performance.

What are the implications of the main findings?

- Wearable sensing may support objective assessment of boxing performance and training.
- Forefoot loading may serve as a practical marker for evaluating kinetic-chain efficiency.

**Abstract:**

Punch velocity is a key performance indicator in boxing and reflects effective coordination along the kinetic chain. This study aimed to investigate the relationship between punch acceleration and plantar pressure distribution using wearable sensing technologies. Twenty-four collegiate boxers (12 professional-level and 12 amateur-level athletes) performed jab and cross punches under controlled conditions. Punch acceleration was measured using a glove-mounted inertial measurement unit (IMU), while plantar pressure distribution was recorded using pressure-sensing insoles. Professional boxers demonstrated significantly higher punch acceleration (22–31%, *p* < 0.05) and greater forefoot plantar pressure (18–27%, *p* < 0.05) compared to amateur athletes. Correlation analysis revealed significant positive associations between forefoot pressure and punch acceleration (r = 0.62–0.71, *p* < 0.01), indicating that increased lower-limb force contributes to higher upper-limb striking performance. These findings demonstrate that combined wearable sensing provides a practical approach for quantifying punching biomechanics and identifying level-dependent kinetic-chain characteristics in boxing.

## 1. Introduction

Among the key performance indicators in boxing, punch velocity is widely recognized as a critical determinant of offensive effectiveness. Previous studies have demonstrated that punching velocity is strongly associated with scoring effectiveness and knockout probability in boxing competition [1,2]. Therefore, accurate quantification of punching performance is essential for both sports science research and practical training applications. From a biomechanical perspective, punching performance is generated through a coordinated kinetic chain involving multiple body segments. Such proximal-to-distal sequencing allows momentum generated by larger body segments to be transferred efficiently to the distal segments of the arm, thereby maximizing punching velocity and impact force [1]. Biomechanical investigations using motion capture systems have demonstrated that trunk rotation velocity and hip extension are critical contributors to punch velocity generation [3]. Similarly, Smith et al. [4] reported that elite boxers exhibit greater trunk angular velocity and improved segmental coordination compared with amateur athletes. These findings highlight the importance of kinetic chain efficiency in determining punching performance.

The movement typically begins with ground reaction forces generated by the lower limbs, which are transmitted through the ankle, knee, and hip joints. Effective coordination within this kinetic chain is essential for maximizing punch speed and impact force. Filimonov et al. [3] demonstrated that elite boxers generate substantial trunk rotational momentum prior to upper limb acceleration, whereas less skilled athletes rely primarily on arm motion. Dunn et al. [2] further showed that variations in trunk rotation timing can significantly influence punching velocity. These findings suggest that distal punch velocity can serve as an important indicator of kinetic chain efficiency during striking movements.

Traditionally, biomechanical analyses of punching techniques have relied on laboratory-based motion capture systems. These systems are considered the gold standard for kinematic analysis because they provide highly accurate measurements of joint angles, segment velocities, and movement trajectories [5]. However, motion capture systems require specialized laboratory environments, expensive equipment, and extensive post-processing procedures, which limit their practical use in daily training settings. As a result, researchers and practitioners have increasingly sought portable and cost-effective alternatives for monitoring athletic performance in real-world environments.

Recent advances in wearable sensor technologies have enabled objective and continuous monitoring of human movement in real-world environments. Wearable sensor technologies have emerged as promising tools for human movement analysis. In particular, inertial measurement units (IMUs), which integrate accelerometers and gyroscopes, have been widely adopted due to their portability, affordability, and ability to capture dynamic motion data outside laboratory environments [6]. In sports science, wearable sensors have been widely applied to quantify movement performance, including velocity, acceleration, force generation, and movement coordination during sport-specific tasks. IMU-based systems have been successfully applied in various sports science applications, including gait analysis, running biomechanics, and skill performance monitoring [7]. In this context, wearable sensors are increasingly adopted as practical tools for in-field biomechanical assessment, where the focus is often on performance evaluation rather than laboratory-based validation. These advantages make IMU-based technologies particularly attractive for monitoring striking performance in combat sports. In combat sports such as boxing, wearable sensing technologies offer a practical approach to capturing punching biomechanics, allowing simultaneous assessment of upper-limb motion and lower-limb force contribution. Specifically, IMU-based motion tracking enables precise measurement of punch acceleration, while plantar pressure sensors provide insight into ground reaction force distribution across different foot regions. The integration of these sensing modalities facilitates a comprehensive understanding of the kinetic chain, linking lower-limb force generation to upper-limb striking performance.

Several studies have explored the use of wearable accelerometers for quantifying punching performance. Kimm and Thiel [8] developed an accelerometer-based approach for estimating hand velocity during boxing movements using triaxial acceleration signals. Similarly, Tiwari et al. [9] attached accelerometers to boxing gloves to analyze punch acceleration characteristics across different striking techniques. Jovanovski and Stappenbelt [10] further combined accelerometers with piezo-resistive sensors to estimate both punch velocity and impact force. Although these studies demonstrate the feasibility of wearable sensors for combat sports analysis, challenges related to sensor placement, measurement reliability, and signal noise remain.

More recent studies have attempted to validate wearable sensor systems for measuring punching velocity. Lambert et al. [11] reported strong correlations between accelerometer-based measurements and linear position transducers when assessing punching velocity. Harris et al. [12] further evaluated the reliability of wearable accelerometers in junior boxing athletes and observed moderate-to-good reliability across repeated trials. Despite these promising findings, variations in measurement accuracy may arise from differences in sensor configuration, sampling frequency, and data processing methods.

Despite increasing interest in wearable sensors for combat sports analysis, several research gaps remain. First, many previous studies have focused primarily on estimating punching velocity without systematically evaluating the reliability of low-cost IMU systems. Second, limited research has investigated the feasibility of using widely accessible consumer-grade sensors for striking performance monitoring. Third, although wearable sensors have been widely used in sports performance monitoring, previous boxing studies have mainly focused on isolated variables such as punch velocity or impact force. Few studies have simultaneously integrated upper-limb acceleration and lower-limb plantar pressure to examine level-dependent kinetic-chain characteristics.

Therefore, the aim of the present study was to investigate the relationship between punch acceleration and plantar pressure distribution in boxers of different competitive levels using wearable sensing technologies. Specifically, this study aimed to examine (1) differences in punch acceleration and plantar pressure between professional and amateur boxers, and (2) the association between lower-limb loading characteristics and upper-limb punching performance.

## 2. Materials and Methods

### 2.1. Subjects

A total of 24 male collegiate boxers were recruited for this study. Based on competitive level and training background, the participants were assigned to a professional group and an amateur group, with 12 athletes in each group. The elite group consisted of boxers who had received formal boxing training for at least 4 years, had achieved top-eight rankings in national collegiate or presidential-level competitions, and were officially registered in the collegiate open division. The amateur group consisted of boxers who had received formal boxing training for less than 1 year, had not obtained county-level or higher competition awards, and were officially registered in the collegiate amateur division. All participants were informed of the experimental procedures before testing and voluntarily agreed to participate. According to the uploaded study materials, the experiment was conducted under an approved ethical framework and included informed consent procedures. To justify sample adequacy for between-group comparisons, a power-based sample size rationale was adopted. Because the primary analyses involved independent-samples comparisons between professional and amateur athletes for punch acceleration and plantar pressure variables, the study was designed to detect practically meaningful group effects. In addition to inferential testing, the current study also aimed to evaluate sensor-derived movement discrimination between competitive levels, which is consistent with exploratory biomechanics studies in combat sports. The descriptive statistics of participant characteristics are presented in Table 1. No significant differences were observed between the two groups in age, height, or body mass. However, the professional group demonstrated substantially longer boxing experience compared with the amateur group. These findings indicate that the two groups were comparable in anthropometric characteristics while differing primarily in training experience and competitive level. The study was conducted in accordance with the Declaration of Helsinki and was approved by the Institutional Research Ethics Review Committee of National Tsing Hua University in Taiwan (11111HT111, 12 January 2023).

### 2.2. Procedures

Commercially available sensing devices were used in this study as measurement tools. The purpose was not to validate the devices themselves, but to use synchronized measurements to investigate biomechanical differences in punching performance. The present study focused on biomechanical comparison and association analysis rather than formal reliability assessment of the measurement devices.

#### 2.2.1. Experimental Overview

Each participant performed two punch types (jab and cross), with two trials per condition. In each trial, five consecutive punches were executed, resulting in a total of 20 punches per participant (2 punch types × 2 trials × 5 punches). To improve data stability and reduce the influence of initial movement variability, the first and last punches of each five-punch trial were excluded, and the middle three punches were retained for analysis. Therefore, a total of 12 valid punches per participant (2 punch types × 2 trials × 3 punches) were included in the final analysis. This trial selection procedure was applied consistently across all participants. Trials were only excluded if clear signal artifacts or execution errors were identified during post-trial inspection; however, no participants required additional exclusion beyond the predefined selection procedure.

**Figure 1:**
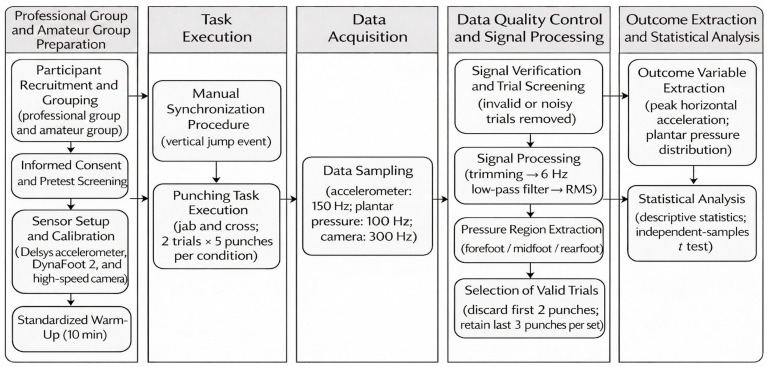
Overview of the experimental workflow used in this study. After participant recruitment and grouping, all subjects completed informed consent, sensor setup, calibration, and standardized warm-up. A vertical jump was used as a manual synchronization event between the accelerometer and plantar pressure systems. Punching trials were then recorded under standardized conditions, followed by data sampling, signal screening, signal processing, valid-trial selection, variable extraction, and statistical analysis.

The overall methodological workflow of the present study is illustrated in Figure 1. After participant recruitment and grouping, all subjects completed pre-test preparation, including informed consent, sensor setup, calibration, and standardized warm-up. A vertical jump event was used to manually synchronize the inertial sensor and plantar pressure systems before formal testing. Punching trials were then acquired under standardized conditions, and all recorded signals subsequently underwent verification, trimming, filtering, and variable extraction prior to statistical analysis.

#### 2.2.2. Instrumentation and Sensor Configuration

Plantar pressure was measured using the DynaFoot 2 dynamic pressure mapping system (Techno Concept, Manosque, France), sampled at 100 Hz. This system was used to quantify plantar pressure distribution during punching and to extract pressure values from the forefoot, midfoot, and rearfoot regions. Punch kinematics were captured using the built-in triaxial accelerometer of the Delsys wireless system (Trigno Standard Sensor, Delsys, Natick, MA, USA). Although the Delsys platform sampled electromyographic signals at 2000 Hz, the built-in accelerometer operated at 150 Hz with a measurement range of ±16 g. The accelerometer sensor was attached to the center of the boxing glove surface in order to quantify peak horizontal acceleration (x-axis) during punching. In addition, a high-speed video camera (CASIO EX-F1, Tokyo, Japan), operating at 300 Hz, was positioned 2 m from the sagittal plane of the participant to record punching execution and to support movement verification during testing. The high-speed camera was used to visually confirm the execution of punching techniques and ensure consistency across trials. The camera was not used for quantitative validation due to differences in measurement modalities and sampling synchronization constraints.

**Figure 2:**
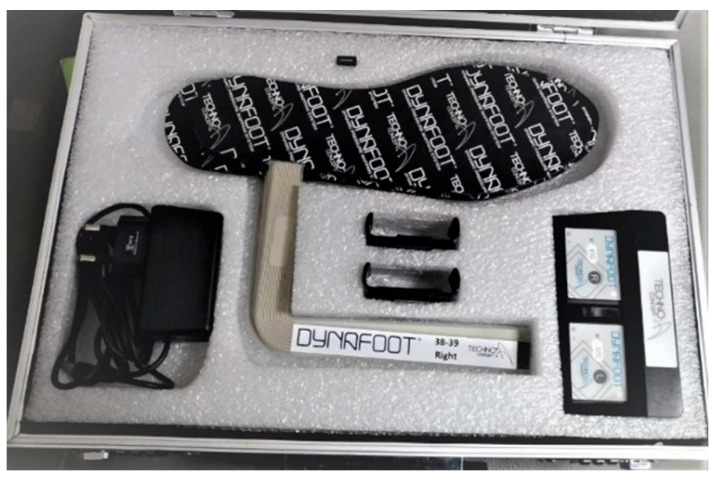
Plantar pressure measurement insole (upper) and wireless signal receiver (below) used in the DynaFoot 2 system. The insole sensor (Techno Concept, Mane, France) was placed inside the shoe to record dynamic plantar pressure during punching, while the receiver was used to collect and transmit the pressure signals for subsequent analysis.

**Figure 3:**
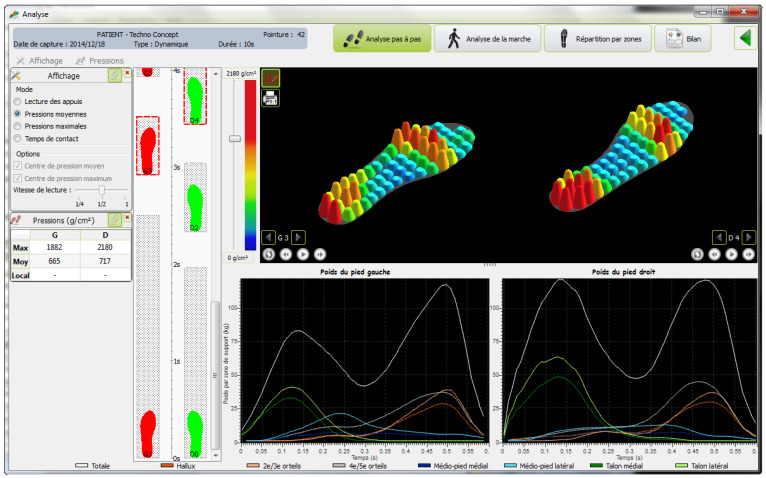
Representative plantar pressure distribution display generated by the DynaFoot 2 system. The interface visualizes regional plantar loading during punching and allows pressure data to be identified in the forefoot, midfoot, and rearfoot regions.

**Figure 4:**
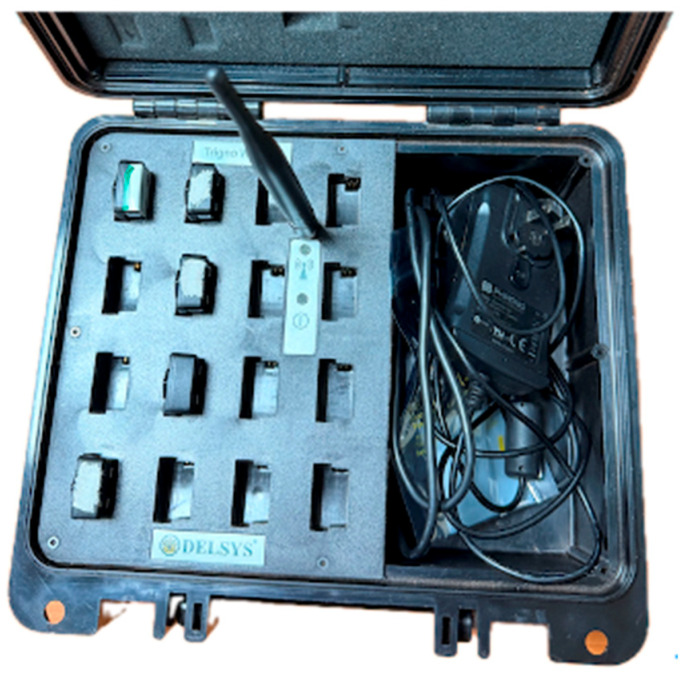
Delsys wireless acquisition system used in this study. The system was employed to record acceleration signals from the built-in triaxial accelerometer during the punching tasks.

**Figure 5:**
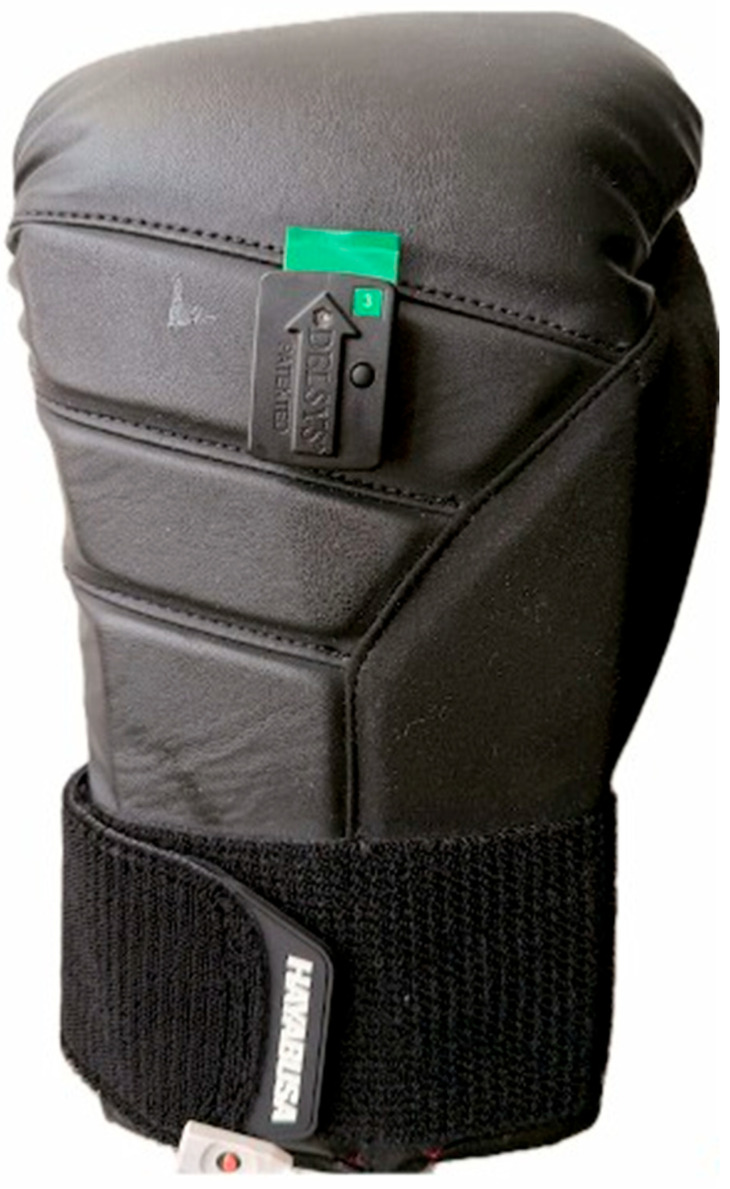
Built-in triaxial accelerometer of the Delsys sensor and its placement on the boxing glove. The sensor was attached to the center of the glove surface to record peak horizontal acceleration (x-axis) during punching.

Punch kinematics were sampled at 150 Hz, plantar pressure data were sampled at 100 Hz, and high-speed video was collected at 300 Hz. These parallel sampling streams enabled simultaneous acquisition of upper-limb acceleration and lower-limb pressure distribution during punching. In inertial-sensor-based sports monitoring, the adequacy of sampling frequency is a critical methodological issue, especially for high-speed striking movements [13,14]. Plantar pressure was measured using the DynaFoot 2 dynamic pressure mapping system (Techno Concept, Mane, France). The plantar pressure insole and wireless receiver used in the experiment are shown in Figure 2. Acceleration signals were collected using the Delsys wireless acquisition system (Figure 3), which includes a built-in triaxial accelerometer for motion measurement. The triaxial accelerometer was attached to the center of the boxing glove surface to capture peak horizontal acceleration during punching (Figure 4). The triaxial accelerometer was attached to the center of the boxing glove surface to capture peak horizontal acceleration during punching (Figure 5).

#### 2.2.3. Sensor Validity and Reliability Rationale

Because the present study relied on a wearable accelerometer and a plantar pressure insole system, sensor validity and reliability were considered during study design. In combat-sport research, inertial sensors have increasingly been used to quantify strike velocity, acceleration, and movement quality, and prior studies have shown that accelerometer-based systems can provide practically useful estimates of punching kinematics when appropriate signal processing and task standardization are applied [6,8,9,10]. The reference Sensors article used a similar engineering rationale, emphasizing that inertial sensing systems are especially valuable when gold-standard motion capture is impractical in field-based sport environments.

For the accelerometer component, the Delsys Trigno platform was selected because of its stable wireless acquisition, synchronized signal output, and consistent sampling architecture. In the present study, the accelerometer was used to extract peak horizontal acceleration (x-axis) rather than full three-dimensional pose reconstruction, thereby reducing model dependency and simplifying interpretation. The use of absolute or peak acceleration features in striking analysis has been adopted in previous combat-sport sensor studies, particularly for high-speed movements in which direct integration and event-based segmentation are practically feasible [8,15]. To improve measurement consistency, sensor placement was fixed at the center of the glove, all punches were performed under standardized conditions, and the same acquisition chain was used for all participants.

For the plantar pressure component, the DynaFoot 2 system was selected because it allows dynamic regional pressure mapping during sport-specific movement. Rather than relying on a single resultant force value, the present protocol analyzed pressure across the forefoot, midfoot, and rearfoot regions, which is more appropriate for investigating lower-limb loading patterns and kinetic-chain initiation during punching. This regional approach improves interpretability of foot–ground interaction and is consistent with the study objective of examining lower-limb contributions to punch generation.

In addition to the literature-based rationale, several procedural steps were implemented to enhance measurement reliability in the present study. These included sensor setup and calibration 30 min before testing, manual synchronization using a vertical jump event, pilot testing before formal data collection, immediate post-trial signal inspection, removal of unstable initial repetitions, and filtering plus software-based signal trimming during post-processing. Together, these procedures were intended to improve within-session consistency and reduce technical noise. This approach is consistent with prior methodological work showing that measurement reliability in explosive and technically demanding movements depends not only on the hardware itself but also on biological variability, technical execution, and test standardization [2,12,16]. To explore the potential applications of wearable device data in training optimization, we conducted correlation analyses to examine the relationship between plantar pressure and punching acceleration. These analyses aimed to assess the feasibility of using biomechanical indicators to guide athletic performance improvement, rather than evaluating the effectiveness of training interventions.

#### 2.2.4. Instrument Calibration and Pre-Test Preparation

All instrumentation was assembled and calibrated 30 min before the participant entered the laboratory. This included installation of the plantar pressure insoles, setup of the Delsys acquisition system, and placement of the high-speed video camera. Signal quality and potential noise interference were checked before data collection began. After entering the laboratory, participants completed a basic information form and received a verbal explanation of the experimental procedures, task requirements, and precautions. Written informed consent was then obtained. All participants performed a standardized 10 min warm-up composed of static and dynamic activities before the formal trials.

#### 2.2.5. Sensor Synchronization and Pilot Testing

To manually synchronize the Delsys accelerometer and the DynaFoot plantar pressure system, each participant performed one vertical jump before the punching trials. This jump was used as a common temporal reference point in both datasets. The take-off and landing phases of the jump were used to align the acceleration and plantar pressure signals before formal data analysis. The use of clearly identifiable synchronization events is consistent with engineering-oriented movement studies in which multiple data streams are aligned in post-processing [8,15]. A pilot test involving five participants was conducted prior to the formal experiment to evaluate protocol feasibility, verify video arrangement, and identify potential problems in signal acquisition and movement execution. These pilot observations were used to refine the formal testing procedure.

#### 2.2.6. Punching Test Procedure

Before data collection, the target position was adjusted individually according to each participant’s punching reach and target height, while maintaining the condition that the glove should not contact the target. During formal testing, participants wore the DynaFoot 2 insoles and boxing gloves equipped with the Delsys accelerometer. They were instructed to perform all punches with maximal effort and maximal speed. Punching frequency was controlled using a metronome set at 60 bpm in order to standardize the temporal structure from the preparatory phase to the acceleration phase. A counterbalanced design was used to reduce order effects. A 5 min rest interval was provided between repeated conditions. To enhance movement consistency and reduce the influence of unstable initial trials, the first two punches in each five-punch set were discarded, and the remaining three punches were used for analysis. The choice of repeated striking trials was based on the need to balance adequate sampling against fatigue and trial-to-trial variability, which are known concerns in explosive sport actions [17,18]. In addition, highly technical movements often show non-negligible within-subject variability, meaning that the first repetition may not always represent the most stable or highest-quality execution [2,16]. Therefore, repeated trials were used to provide a more robust basis for variable extraction.

#### 2.2.7. Data Verification and Signal Processing

After each test session, the data were checked to ensure that the following variables had been successfully collected: (1) maximal plantar pressure values in the forefoot, midfoot, and rearfoot regions; and (2) peak horizontal acceleration (x-axis) during the jab and cross from the preparatory phase to the acceleration phase. If excessive signal noise or recording errors were observed, the corresponding trial was repeated. Acceleration signals were processed in EMGworks analysis software. The raw acceleration traces were first trimmed to isolate the punching interval from the preparatory phase to the acceleration phase. The selected signals were then low-pass-filtered with a cutoff frequency of 6 Hz, and the root mean square (RMS) procedure was applied to derive the acceleration variable in m/s^2^. The selection of the cutoff frequency was based on established biomechanical signal processing methods, which recommend determining an optimal cutoff frequency to minimize noise while preserving movement-related signal characteristics [19]. The acceleration signals were processed using a fourth-order Butterworth low-pass filter with a cutoff frequency of 6 Hz. Zero-phase filtering was implemented using a forward–backward filtering approach to avoid phase distortion. The cutoff frequency was selected to balance noise reduction and signal preservation, consistent with prior studies on human movement analysis. Previous studies have demonstrated that cutoff frequencies within the range of 5–20 Hz are commonly used for human motion and IMU-based biomechanical data, depending on movement dynamics and sampling frequency [20]. In addition, residual analysis and optimization approaches have been proposed to determine appropriate cutoff frequencies for minimizing signal distortion in kinematic data [19]. Plantar pressure data were exported from the DynaFoot software (Version 2) into Microsoft Excel for preprocessing and organization prior to statistical analysis. Acceleration data were analyzed along the primary punching direction, defined as the forward horizontal axis relative to the glove orientation. The coordinate system of the IMU was aligned such that the x-axis corresponded to the forward punching direction, the y-axis to the lateral direction, and the z-axis to the vertical direction. Gravity correction was applied by subtracting the static gravitational component from the raw acceleration signal prior to further processing. The IMU coordinate system was defined relative to the glove orientation. The x-axis corresponded to the forward punching direction, the y-axis represented the mediolateral direction, and the z-axis represented the vertical direction. Only the x-axis component was used for further analysis as it aligns with the primary punching motion.

Signal processing choices were informed by prior inertial-sensor studies that used event-based segmentation, absolute or resultant acceleration features, and numerical or smoothed post-processing for high-speed strikes [8,15]. More broadly, smartphone- and IMU-based motion studies have emphasized that signal reliability depends not only on the hardware but also on the post-processing chain, including filtering, trimming, and noise management [7,14,21]. For this reason, the present study incorporated both immediate post-trial screening and offline software-based processing to improve signal stability before statistical comparison. Due to limitations in exporting raw waveform and spatial pressure maps, data visualization was performed using detailed tabular summaries of temporal and regional characteristics.

Acceleration variables were defined as follows: (1) Peak acceleration (x-axis): the maximum acceleration value along the forward punching direction. (2) RMS acceleration (x-axis): the root mean square of acceleration along the x-axis, representing the overall magnitude of the punching motion over time. (3) Mean horizontal acceleration (x-axis): the average acceleration over the punch duration after filtering. Differences in magnitude between these variables are expected, as peak values represent instantaneous maxima, whereas RMS and mean values reflect time-averaged signal characteristics.

#### 2.2.8. Multi-Modal Measurement Data and Classification

To further explore the combined effect of upper- and lower-limb variables, a multi-modal measurement data approach was applied by integrating punch acceleration and plantar pressure features into composite indicators. Specifically, normalized forefoot plantar pressure and peak punch acceleration were combined to form a performance index. In addition, a classification analysis was performed to evaluate the ability of the fused variables to distinguish between professional and amateur boxers. A logistic regression model was used, with punch acceleration and plantar pressure variables as predictors and athlete level as the dependent variable.

### 2.3. Statistical Analysis

All statistical analyses were performed using SPSS 21.0. Descriptive statistics were first used to summarize participant characteristics in the professional and amateur groups. Table 1 presents the basic characteristics of the two groups. Independent-samples *t*-tests were then conducted to compare the two groups with respect to peak horizontal acceleration (x-axis) during the jab and cross, as well as plantar pressure variables in the forefoot, midfoot, and rearfoot regions of both the lead and rear legs. The level of significance was set at α = 0.05.

Although the present study focused on between-group differences rather than repeated-session reliability, the statistical rationale was informed by methodological literature commonly cited in sensor-based movement studies. In particular, Atkinson and Nevill [22] and Hopkins [23] emphasized the importance of considering measurement variability and error structure when interpreting sport-performance data. Similarly, Shrout and Fleiss [24] provided the standard framework for intraclass correlation coefficient models that are often used in sensor reliability studies, including the reference Sensors paper. In the current study, inferential statistics were limited to independent-samples comparisons because the primary objective was to determine whether wearable-sensor-derived variables could discriminate between competitive levels under a standardized testing protocol.

To account for repeated measurements within participants, a linear mixed-effects model was used instead of multiple independent *t*-tests. Punch type (jab vs. cross), foot (lead vs. rear), and region (forefoot, midfoot, rearfoot) were treated as fixed effects, while subject was included as a random effect to account for within-subject variability. Group (professional vs. amateur) was included as a between-subject factor. Interaction effects were also examined where appropriate. Post hoc comparisons were conducted with Bonferroni correction to control for multiple comparisons.

## 3. Results

The comparison of mean horizontal acceleration between the professional and amateur groups is presented in Table 2. Independent samples *t*-tests revealed significant differences in both jab and straight punches. The professional group demonstrated significantly greater acceleration during jab punching (*t* = 2.97, *p* = 0.007), with a large effect size (Cohen’s d = 1.23, 95% CI [0.25, 2.18]). Similarly, the straight punch showed significantly higher acceleration in the professional group (*t* = 3.30, *p* = 0.003), also representing a large effect size (d = 1.35, 95% CI [0.36, 2.32]). These results indicate that professional-level boxers are capable of producing greater punching acceleration during straight-line punching actions.

The linear mixed-effects analysis revealed a significant main effect of group on punch acceleration (*p* < 0.001) (Table 3), with professional athletes demonstrating higher values than amateurs. Significant effects were also observed for punch type *(p* < 0.01) and plantar region (*p* < 0.01), indicating that both movement type and foot loading characteristics influence punching performance. A significant interaction between group and plantar region (*p* < 0.05) was identified, suggesting that professional athletes exhibit a more forefoot-dominant loading pattern compared to amateurs. Post hoc comparisons with Bonferroni correction confirmed that forefoot pressure was significantly higher in professional athletes across both punch types.

The plantar pressure distribution during jab punching is summarized in Table 4. Significant differences were observed in the lead foot plantar pressure between the two groups. The professional group exhibited significantly greater pressure in the forefoot region of the lead foot (*p* = 0.001, d = 2.98). In contrast, the amateur group demonstrated greater pressure in the rearfoot region (*p* = 0.028, d = 1.12). No significant differences were observed in the midfoot region or in any region of the rear foot.

The plantar pressure results during straight punching are presented in Table 5. Significant differences were observed in the forefoot region of both feet. The professional group demonstrated significantly higher plantar pressure in the forefoot of the lead foot (*p* = 0.045, d = 1.10) and rear foot (*p* = 0.042, d = 1.12). No significant differences were observed in the midfoot or rearfoot regions.

Correlation analyses revealed significant associations between plantar pressure variables and punch acceleration (Table 6). Specifically, lead foot forefoot plantar pressure was moderately correlated with jab peak acceleration (r = 0.62, *p* < 0.01), indicating that greater force generation in the lead foot contributes to higher jab performance. For the cross punch, rear foot forefoot pressure demonstrated a strong positive correlation with peak acceleration (r = 0.71, *p* < 0.001), highlighting the critical role of rear-leg propulsion in generating higher striking velocity. Total plantar pressure was also significantly correlated with both jab (r = 0.57, *p* < 0.01) and cross acceleration (r = 0.63, *p* < 0.01). In contrast, midfoot and rearfoot pressure variables showed weak and non-significant correlations with punch acceleration, suggesting that forefoot-dominant force generation plays a more prominent role in punching biomechanics.

The multi-modal measurement data analysis demonstrated that the combined use of punch acceleration and plantar pressure improved the differentiation between professional and amateur athletes compared to single-variable analysis (Table 7). The classification model achieved an accuracy of 83.3%, with a sensitivity of 0.83 and specificity of 0.84. Punch acceleration and forefoot plantar pressure were identified as significant predictors (*p* < 0.05). These results indicate that integrating upper- and lower-limb variables provides a more comprehensive representation of boxing performance.

To further examine inter-individual variability, individual-level data were analyzed for both punch acceleration and plantar pressure variables (Table 8). Considerable variability was observed within each group, with professional boxers demonstrating not only higher mean values but also more consistent performance patterns compared to amateur athletes. Specifically, the coefficient of variation (CV) for punch acceleration was lower in professional boxers (CV = 8.5%) than in amateurs (CV = 14.2%), indicating more stable neuromuscular control. Similarly, forefoot plantar pressure showed reduced variability in professionals, suggesting more consistent lower-limb force generation strategies. These findings highlight the importance of considering individual differences in biomechanical performance, beyond group-level comparisons.

To enhance the interpretability of the results without graphical representations, detailed tabular summaries of dynamic characteristics and plantar pressure distribution were provided. Temporal characteristics of punch acceleration (Table 9) showed that professional boxers achieved higher peak acceleration with shorter time-to-peak values compared to amateurs, indicating more explosive movement patterns. Regional plantar pressure distribution (Table 10) revealed a forefoot-dominant loading strategy in professional athletes, whereas amateur boxers exhibited greater rearfoot reliance. Correlation analysis (Table 11) further demonstrated a significant positive relationship between forefoot pressure and punch acceleration, highlighting the role of lower-limb force generation in punching performance. It should be noted that the acceleration values reported in different tables represent distinct signal metrics. The values in Table 2 correspond to mean acceleration over time, whereas Table 9 reports peak and RMS acceleration values. As expected, peak acceleration values are substantially higher than mean values, as they capture instantaneous maxima of the signal, while RMS values reflect the overall signal magnitude. These differences explain the variation in magnitude across tables.

**Figure 6:**
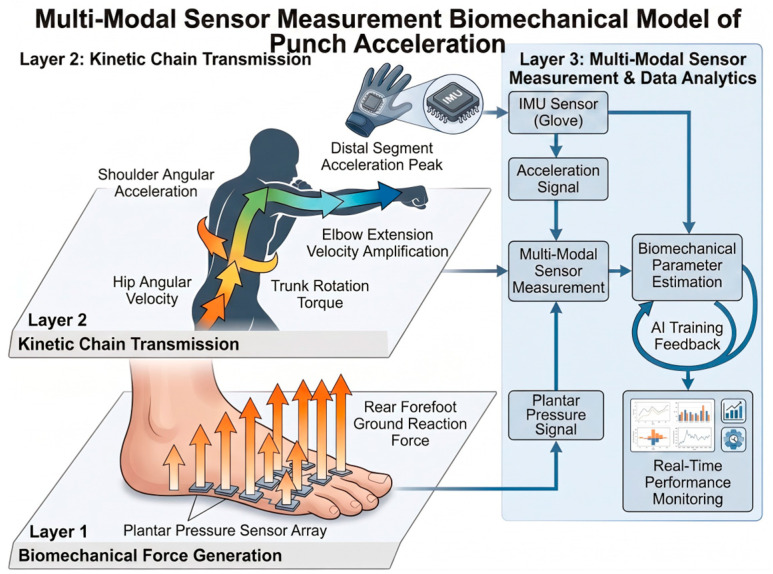
Conceptual model illustrating the relationship between plantar pressure and punch acceleration during straight punching. Ground reaction force generated through rear forefoot drive initiates sequential kinetic chain transmission involving hip rotation, trunk rotation, shoulder acceleration, and elbow extension, resulting in maximal distal segment acceleration at the fist. The model summarizes the quantitative relationship observed in the present study and highlights the role of wearable sensors in capturing lower-limb force generation and upper-limb motion for real-time biomechanical monitoring and performance analysis.

Overall, the professional group demonstrated significantly greater punching acceleration in both jab and straight punches compared with the amateur group. In terms of plantar pressure distribution, professional boxers exhibited a forefoot-dominant force application pattern. During jab punching, the lead-foot forefoot acted as the primary propulsion region, whereas amateur boxers relied more on the rearfoot. During straight punching, both the lead- and rear-foot forefoot regions showed significantly greater pressure in the professional group, suggesting a coordinated bilateral forefoot-driven kinetic chain mechanism. To summarize the quantitative relationship between plantar pressure and punch acceleration observed in the present study, a conceptual kinetic chain model is presented in Figure 6. The model illustrates how forefoot-dominant force generation contributes to sequential energy transfer and distal acceleration amplification.

## 4. Discussion

It should be noted that the present study did not aim to establish measurement reliability in a methodological sense, but rather to examine biomechanical differences and associations derived from wearable sensor data. The observed differences between mean, peak, and RMS acceleration values reflect the different signal processing approaches used to characterize punching dynamics, rather than inconsistencies in the measurements themselves. The present study examined differences in punch acceleration and plantar pressure distribution between professional and amateur boxers during jab and straight punches. The use of a mixed-effects model allowed us to account for repeated measurements within subjects and provided a more robust statistical framework compared to multiple independent *t*-tests. This approach reduces the risk of inflated Type I error and better reflects the hierarchical structure of the data. The results revealed that professional athletes generated significantly higher horizontal punch acceleration in both punching techniques. These findings indicate that professional athletes demonstrate more efficient neuromuscular coordination and kinetic chain transmission during punching movements. The observed inter-individual variability provides additional insight into the neuromuscular coordination strategies of boxers. Professional athletes exhibited not only higher performance outputs but also reduced variability, which may reflect more refined motor control and consistent kinetic chain activation. In contrast, greater variability in amateur athletes may indicate less stable coordination between lower- and upper-limb segments. These findings suggest that performance assessment should consider both magnitude and consistency of biomechanical variables.

Punching performance in combat sports is strongly influenced by the interaction between lower-limb force production and upper-limb motion. Previous studies have demonstrated that effective lower-body drive contributes significantly to punching velocity and impact force [25,26]. Turner et al. reported that increased ground reaction forces from the lower extremities can significantly enhance rear-hand punch impact force [26].

Biomechanically, punch velocity is produced through a proximal-to-distal sequence of segmental motion, where force generated from the lower limbs is transmitted through trunk rotation to the upper limbs [27]. Dinu et al. reported that professional boxers produce higher trunk angular velocities and elbow extension speeds during punching, which contribute significantly to punch velocity [28]. Therefore, the greater punch acceleration observed in professional athletes likely reflects more efficient kinetic chain coordination.

**Figure 7:**
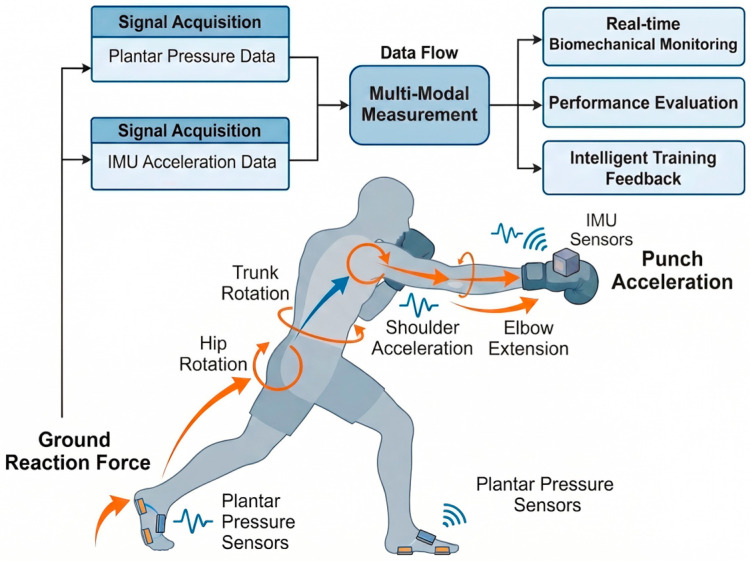
Conceptual model of sensor-based punching biomechanics illustrating the proximal-to-distal kinetic chain during straight punching. Ground reaction force generated at the rear forefoot initiates hip and trunk rotation, followed by shoulder acceleration and elbow extension, resulting in maximal punch acceleration at the distal segment. The model highlights the biomechanical mechanism underlying punch performance and the role of wearable sensors in capturing lower-limb force generation and upper-limb motion.

To further interpret the biomechanical mechanism underlying the present findings, a conceptual kinetic chain model of punching is illustrated in Figure 7. The model demonstrates that punch acceleration is primarily generated through forefoot-dominant ground reaction forces, followed by sequential energy transfer from the lower limbs to the trunk and upper extremity. The conceptual model presented in the figure illustrates the multi-modal measurement underlying punch acceleration during straight punching. The movement is initiated by ground reaction forces generated at the rear forefoot, which serve as the primary source of force production. This force is transmitted proximally through the lower limbs, resulting in increased hip angular velocity and subsequent trunk rotational torque. The sequential transfer of mechanical energy continues through the shoulder joint, leading to upper-limb acceleration, followed by rapid elbow extension that amplifies distal segment velocity. As a result of this proximal-to-distal kinetic chain transmission, maximal punch acceleration is achieved at the fist.

In parallel, wearable inertial measurement units positioned at the glove capture upper-limb acceleration signals, while plantar pressure sensors embedded in the insole quantify lower-limb force generation and ground contact characteristics. These biomechanical and kinetic signals are integrated through a sensor multi-modal measurement, enabling real-time estimation of punching performance variables. Furthermore, the proposed model highlights the potential application of wearable sensing technologies for intelligent training systems, movement monitoring, and human health management by providing objective feedback on neuromuscular coordination and kinetic chain efficiency. This model provides a theoretical basis for understanding how forefoot-dominant force generation contributes to the observed increases in punch acceleration among higher-level athletes.

Plantar pressure analysis revealed significant differences between groups in the forefoot regions during punching. The professional group exhibited greater plantar pressure in the forefoot region during both jab and straight punches. Plantar pressure distribution reflects the location and magnitude of ground reaction force generation during athletic movements. These findings suggest that forefoot force generation plays a critical role in initiating the kinetic chain during punching. Professional boxers appear to adopt a forefoot-dominant strategy when generating punching force. This strategy facilitates forward body momentum and efficient transmission of force from the lower limbs to the upper body. In contrast, amateur athletes often rely more on rearfoot loading, which may reduce the efficiency of forward force transfer and limit punch acceleration.

Recent advances in wearable sensing technologies enable the integration of multiple sensor modalities to analyze complex athletic movements. In the present study, punch acceleration measured using inertial sensors was combined with plantar pressure measurements to examine the biomechanical characteristics of punching. Wearable inertial measurement units (IMUs) have been widely used to quantify movement velocity and acceleration in sports biomechanics [8]. Kimm and Thiel demonstrated that IMU sensors can reliably measure punching velocity in boxing [8]. Similarly, Stanley et al. reported that lower-limb kinetics play a crucial role in punch performance using three-dimensional biomechanical analysis [29]. Combining IMU sensors with plantar pressure systems enables the development of a sensor multi-modal measurement, where upper-limb motion and lower-limb force generation can be measured simultaneously. Such a framework provides a more comprehensive understanding of the kinetic chain mechanisms involved in punching movements. The proposed multi-modal measurement data approach demonstrates the potential for integrating multiple wearable sensor signals to enhance performance assessment in boxing. It should be noted that the present study does not implement a formal sensor fusion algorithm. Instead, the analysis is based on the integration of variables derived from multiple wearable sensors to examine biomechanical relationships. Although a full expert system was not implemented in this study, the classification results indicate that fused biomechanical variables can effectively distinguish athlete skill levels. These findings provide a foundation for future development of intelligent training systems, where multi-modal sensor data can be used to automatically evaluate performance and support decision-making in coaching and athlete development.

Wearable sensor technologies are increasingly used in human health monitoring and sports performance analysis. Sensors capable of measuring movement patterns and force distribution can provide valuable insights into injury prevention, rehabilitation, and athletic performance. Camomilla et al. discussed the growing application of wearable inertial sensors in sports biomechanics [30]. Peake et al. emphasized the role of wearable technologies in monitoring athlete workload and health status [31]. Similarly, Yang et al. demonstrated the feasibility of wearable sensor systems for human motion monitoring in sports and rehabilitation settings [32]. Recent advances in wearable sensor technologies have enabled objective and continuous monitoring of human movement and physiological signals in real-world environments. Emerging soft, skin-interfaced, and textile-integrated sensing systems have demonstrated capabilities in continuous blood pressure monitoring, respiration tracking, and wireless physiological sensing, significantly expanding the scope of wearable technologies in human health and performance applications [33,34,35,36,37,38,39,40]. In combat sports such as boxing, wearable sensing technologies offer a practical approach to capturing punching biomechanics, allowing simultaneous assessment of upper-limb motion and lower-limb force contribution. Specifically, IMU-based motion tracking enables precise measurement of punch acceleration, while plantar pressure sensors provide insight into ground reaction force distribution across different foot regions. The integration of these sensing modalities facilitates a comprehensive understanding of the kinetic chain, linking lower-limb force generation to upper-limb striking performance. Despite these advancements, existing studies have often focused on isolated measurements, lacking a unified sensor multi-modal measurement that simultaneously captures punch acceleration and plantar pressure distribution. Therefore, this study proposes a wearable sensor multi-modal measurement approach combining glove-mounted IMUs and pressure-sensing insoles to quantify punching biomechanics and investigate performance differences between athlete levels.

Although the present study did not include a longitudinal training intervention, the observed relationships between plantar pressure and punch acceleration provide important insights into potential training optimization strategies. Specifically, the strong association between forefoot loading and punching performance suggests that training programs focusing on lower-limb force generation and weight transfer may enhance punching effectiveness. These findings support the potential use of wearable sensor-derived variables as objective indicators for monitoring and guiding training adaptations. Future studies should incorporate longitudinal intervention designs to validate the effectiveness of such training strategies.

Although a high-speed camera was used in this study, it served primarily as a qualitative tool for motion verification rather than a quantitative reference for validation. Previous studies have demonstrated that IMU-based measurements provide valid and reliable estimates of high-speed limb movements in sports applications, particularly for acceleration and velocity assessment [5,6,30]. Therefore, the present study focused on the application of wearable sensing technologies for biomechanical comparison rather than device validation. Future studies are encouraged to incorporate synchronized high-speed motion capture systems to further validate multi-modal measurements and enhance the accuracy of biomechanical assessments.

## 5. Conclusions

This study investigated punch acceleration and plantar pressure distribution in straight punching techniques (jab and cross) among boxers of different competitive levels using wearable inertial measurement units and plantar pressure sensors. The results demonstrated that professional boxers produced significantly greater horizontal punch acceleration than amateur boxers in both punching techniques, indicating superior neuromuscular coordination and more efficient kinetic chain transmission. In terms of plantar pressure distribution, professional athletes exhibited higher forefoot pressure in the lead foot during the jab and greater forefoot pressure in both feet during the cross. These findings support the proposed kinetic chain model in which force generation begins with forefoot-dominant ground reaction forces, followed by energy transfer through the lower limbs, trunk rotation, and upper-limb acceleration, ultimately producing greater punching velocity. The findings indicate that wearable IMU and plantar pressure systems can serve as feasible tools for analyzing punching biomechanics and identifying kinetic-chain-related differences between boxers of different competitive levels. Such wearable sensing technologies allow simultaneous monitoring of lower-limb force generation and upper-limb motion, offering valuable applications in sports performance evaluation and movement monitoring. From a broader perspective, sensor-based biomechanical monitoring may facilitate early detection of inefficient movement patterns and contribute to training optimization. Future studies should further investigate punching biomechanics under dynamic conditions such as footwork, defensive actions, and fatigue states, and expand wearable sensor applications for long-term monitoring of athlete performance.

## Figures and Tables

**Table 1:** Participant characteristics.

Variable	Professional (*n* = 12)	Amateur (*n* = 12)
Age (years)	21.92 ± 1.73	21.83 ± 1.90
Height (cm)	173.75 ± 3.44	172.92 ± 4.10
Body mass (kg)	66.75 ± 6.28	67.42 ± 7.90
Boxing experience (years)	6.50 ± 1.51	1.60 ± 0.78

**Table 2:** Mean horizontal acceleration (x-axis) during punching (m/s^2^).

Punch Type	Professional (m/s^2^)	Amateur (m/s^2^)	*t*-Value	*p*-Value	Cohen’s d	95% CI
Jab	1.96 ± 0.81	1.22 ± 0.30	2.97	0.007 **	1.23	[0.25, 2.18]
Straight	2.19 ± 0.69	1.39 ± 0.47	3.30	0.003 **	1.35	[0.36, 2.32]

* *p* < 0.05, ** *p* < 0.01

**Table 3:** Linear mixed-effects model results for punch acceleration and plantar pressure.

Effect	F-Value	*p*-Value
Group	12.45	<0.001 ***
Punch type	6.32	0.014 *
Region	8.71	0.002 **
Group × Region	4.25	0.038 *

* *p* < 0.05, ** *p* < 0.01, *** *p* < 0.001.

**Table 4:** Plantar pressure distribution during jab punching.

Foot	Region	Professional (N/cm^2^)	Amateur (N/cm^2^)	*p*-Value	Cohen’s d
Lead	Forefoot	19.65 ± 2.79	8.79 ± 2.26	<0.001 ***	2.98
Lead	Midfoot	7.21 ± 3.31	11.03 ± 4.03	0.057	0.96
Lead	Rearfoot	8.59 ± 4.03	12.35 ± 1.62	0.028 *	1.12
Rear	Forefoot	13.80 ± 7.33	9.97 ± 4.18	0.220	0.62
Rear	Midfoot	8.59 ± 4.96	10.48 ± 3.69	0.405	0.44
Rear	Rearfoot	11.03 ± 6.69	9.31 ± 4.32	0.548	0.30

* *p* < 0.05, *** *p* < 0.001.

**Table 5:** Plantar pressure distribution during straight punching.

Foot	Region	Professional (N/cm^2^)	Amateur (N/cm^2^)	*p*-Value	Cohen’s d
Lead	Forefoot	12.37 ± 3.04	9.26 ± 2.57	0.045 *	1.10
Lead	Midfoot	7.74 ± 4.12	9.01 ± 2.84	0.487	0.36
Lead	Rearfoot	7.83 ± 6.01	6.96 ± 4.79	0.753	0.16
Rear	Forefoot	12.28 ± 3.09	9.18 ± 2.42	0.042 *	1.12
Rear	Midfoot	7.48 ± 3.71	9.01 ± 2.84	0.371	0.46
Rear	Rearfoot	7.76 ± 5.66	7.14 ± 4.87	0.816	0.11

* *p* < 0.05.

**Table 6:** Correlation coefficients between plantar pressure variables and punch acceleration.

Variable	Jab Acceleration	Cross Acceleration
Lead foot forefoot pressure	0.62 **	0.48 *
Rear foot forefoot pressure	0.45 *	0.71 ***
Midfoot pressure	0.21	0.26
Rearfoot pressure	0.18	0.22
Total plantar pressure	0.57 **	0.63 **

* *p* < 0.05, ** *p* < 0.01, *** *p* < 0.001.

**Table 7:** Classification performance based on fused biomechanical variables.

Model	Accuracy	Sensitivity	Specificity
Acceleration only	70.8%	0.71	0.70
Pressure only	66.7%	0.65	0.68
Multi-modal measurement model	83.3%	0.83	0.84

**Table 8:** Inter-individual variability metrics.

Variable	Group	CV (%)
Jab acceleration	Professional	8.5
	Amateur	14.2
Forefoot pressure	Professional	9.1
	Amateur	15.6

**Table 9:** Peak and RMS acceleration values (x-axis) during punching.

Variable	Professional (*n* = 12)	Amateur (*n* = 12)	*p*-Value
Peak acceleration (m/s^2^)	21.2 ± 2.8	16.3 ± 2.5	<0.001 ***
Time to peak (ms)	120 ± 15	145 ± 18	0.003 **
RMS acceleration (m/s^2^)	14.8 ± 2.1	11.2 ± 1.9	0.002 **

** *p* < 0.01, *** *p* < 0.001.

**Table 10:** Regional plantar pressure distribution (% body weight).

Region	Professional (*n* = 12)	Amateur (*n* = 12)	*p*-Value	Cohen’s d
Forefoot	62.4 ± 5.1	50.8 ± 6.2	<0.001 ***	2.05
Midfoot	21.3 ± 3.8	23.1 ± 4.2	0.312	0.45
Rearfoot	16.3 ± 3.1	26.1 ± 4.5	0.004 **	1.98

** *p* < 0.01, *** *p* < 0.001.

**Table 11:** Correlation between plantar pressure and punch acceleration.

Variable	r	*p*-Value
Forefoot pressure vs. acceleration	0.62	0.004 **
Rearfoot pressure vs. acceleration	−0.48	0.021 *
Total pressure vs. acceleration	0.63	0.002 **

* *p* < 0.05, ** *p* < 0.01

## Data Availability

The datasets used and analyzed during the current study are available from the corresponding author on reasonable request.

## Abbreviations

CI	Confidence Interval
RMS	Root Mean Square
